# Supplementary material for: PheGWAS: a new dimension to visualize GWAS across multiple phenotypes
Source: Bioinformatics. 2019 Dec 20;36(8):2500–5. doi: 10.1093/bioinformatics/btz944 (PMC7178436; doi:10.1093/bioinformatics/btz944)
Supplement: btz944_Supplementary_Data [file btz944_supplementary_data.pdf]

TABLES:

Supplementary Table 1a: Comparative analysis loci identified by GLGC and PheGWAS results for association of loci with single traits

Nearest Gene	SNP ID	Chromosome	Position MB	Trait	In PheGWAS	Gene Match	Identifying GLGC SNP's
EVI5	rs7515577	1	93.01	TC	DETECTED	M	B
ZNF648	rs1689800	1	182.17	HDL	DETECTED	NM	B
PABPC4	rs4660293	1	40.03	HDL	DETECTED	M	A
ASAP3	rs1077514	1	23.77	TC	DETECTED	M	A
ANXA9	rs267733	1	150.96	LDL	DETECTED	M	A
RRNAD1	rs12145743	1	156.70	HDL	DETECTED	M	A
C1orf220	rs4650994	1	178.52	HDL	DETECTED	M	A
RAB3GAP1	rs7570971	2	135.84	TC	DETECTED	M	B
COBLL1	rs12328675	2	165.54	HDL	DETECTED	M	B
EHBP1	rs2710642	2	63.15	LDL	DETECTED	M	A
ABCB11	rs2287623	2	169.83	TC	DETECTED	M	A
FAM117B	rs11694172	2	203.53	TC	DETECTED	M	A
CPS1	rs1047891	2	211.54	HDL	DETECTED	M	A
FN1	rs1250229	2	216.30	LDL	DETECTED	M	A
RAF1	rs2290159	3	12.63	TC	DETECTED	NM	C (level 3)
MSL2L1	rs645040	3	135.93	TG	DETECTED	M	A
ATG7	rs2606736	3	11.40	HDL	DETECTED	M	A
SETD2	rs2290547	3	47.06	HDL	DETECTED	M	A
RBM5	rs2013208	3	50.13	HDL	DETECTED	M	A
STAB1	rs13326165	3	52.53	HDL	DETECTED	M	A
PXK	rs13315871	3	58.38	TC	DETECTED	M	A
GSK3B	rs6805251	3	119.56	HDL	DETECTED	M	A
KLHL8	rs442177	4	88.03	TG	DETECTED	NM	A
SLC39A8	rs13107325	4	103.19	HDL	DETECTED	M	A
C4orf52	rs10019888	4	26.06	HDL	DETECTED	NA	A
FAM13A	rs3822072	4	89.74	HDL	DETECTED	M	A
ADH5	rs2602836	4	100.01	HDL	DETECTED	M	A
ARL15	rs6450176	5	53.30	HDL	DETECTED	M	A
MAP3K1	rs9686661	5	55.86	TG	DETECTED	NM	A
CITED2	rs605066	6	139.83	HDL	DETECTED	NM	B
C6orf106	rs2814982	6	34.55	TC	DETECTED	M	A
KCNK17	rs2758886	6	39.25	TC	DETECTED	M	A
HBS1L	rs9376090	6	135.41	TC	DETECTED	M	A
TYW1B	rs13238203	7	72.13	TG	DETECTED	NM	B
KLF14	rs4731702	7	130.43	HDL	DETECTED	M	B
GPR146	rs1997243	7	1.08	TC	DETECTED	M	A
DAGLB	rs702485	7	6.45	HDL	DETECTED	M	A
SNX13	rs4142995	7	17.92	HDL	DETECTED	M	A
IKZF1	rs4917014	7	50.31	HDL	DETECTED	M	A
MET	rs38855	7	116.36	TG	DETECTED	M	A
TMEM176A	rs17173637	7	150.53	HDL	DETECTED	M	A
PINX1	rs11776767	8	10.68	TG	DETECTED	M	B
TRPS1	rs2293889	8	116.60	HDL	DETECTED	M	A
JMJD1C	rs10761731	10	65.03	TG	DETECTED	M	B
CYP26A1	rs2068888	10	94.84	TG	DETECTED	M	A
AKR1C4	rs1832007	10	5.25	TG	DETECTED	M	A
VIM	rs10904908	10	17.26	TC	DETECTED	M	A
SPTY2D1	rs10128711	11	18.63	TC	DETECTED	M	B
AMPD3	rs2923084	11	10.39	HDL	DETECTED	M	A
LRP4	rs3136441	11	46.74	HDL	DETECTED	NM	A
OR4C46	rs11246602	11	51.51	HDL	DETECTED	M	A
PCNXL3	rs12801636	11	65.39	HDL	DETECTED	M	A
MOGAT2	rs499974	11	75.46	HDL	DETECTED	M	A
PHLDB1	rs11603023	11	118.49	TC	DETECTED	M	A
PDE3A	rs7134375	12	20.47	HDL	DETECTED	NA	B
MVK	rs7134594	12	110.00	HDL	DETECTED	M	B
SBNO1	rs4759375	12	123.80	HDL	DETECTED	M	C (level 1)
SCARB1	rs838880	12	125.26	HDL	DETECTED	M	B
PHC1	rs4883201	12	9.08	TC	DETECTED	M	A
BRCA2	rs4942486	13	32.95	LDL	DETECTED	M	A
NYNRIN	rs8017377	14	24.88	LDL	DETECTED	M	A
ZBTB42	rs4983559	14	105.28	HDL	DETECTED	M	A
FRMD5	rs2929282	15	44.25	TG	DETECTED	M	B
CAPN3	rs2412710	15	42.68	TG	DETECTED	M	A
LACTB	rs2652834	15	63.40	HDL	DETECTED	M	A
CTF1	rs11649653	16	30.92	TG	DETECTED	M	A
LCAT	rs16942887	16	67.93	HDL	DETECTED	M	A
CMIP	rs2925979	16	81.53	HDL	DETECTED	M	A
PDXDC1	rs3198697	16	15.13	TG	DETECTED	M	A
STARD3	rs11869286	17	37.81	HDL	DETECTED	M	B
PGS1	rs4129767	17	76.40	HDL	DETECTED	M	B
ABCA8	rs4148008	17	66.88	HDL	DETECTED	M	A
MPP3	rs8077889	17	41.88	TG	DETECTED	M	A
APOH	rs1801689	17	64.21	LDL	DETECTED	M	A
MC4R	rs12967135	18	57.85	HDL	DETECTED	NM	B
ANGPTL4	rs7255436	19	8.43	HDL	DETECTED	M	B
FLJ36070	rs492602	19	49.21	TC	DETECTED	NM	B
LILRA3	rs386000	19	54.79	HDL	DETECTED	M	B
ANGPTL8	rs737337	19	11.35	HDL	DETECTED	NM	A
INSR	rs7248104	19	7.22	TG	DETECTED	M	A
FPR3	rs17695224	19	52.32	HDL	DETECTED	M	A
ERGIC3	rs2277862	20	34.15	TC	DETECTED	M	B
SPTLC3	rs364585	20	12.96	LDL	DETECTED	M	A
SNX5	rs2328223	20	17.85	LDL	DETECTED	NM	A
UBE2L3	rs181362	22	21.93	HDL	DETECTED	M	C (level 1)
PLA2G6	rs5756931	22	38.55	TG	DETECTED	M	B
MTMR3	rs5763662	22	30.38	LDL	DETECTED	M	A
TOM1	rs138777	22	35.71	TC	DETECTED	M	A

In this table, the columns in pheGWAS, gene match and SNP match corresponds to the PheGWAS results we obtained.

Match (M); if the gene symbol in a particular PheGWAS base pair interval segment is the same trait as reported in the GLGC
No Match (NM); if the gene is not identified by PheGWAS.

A- SNP identical with GLGC and PheGWAS
B – GLGC SNP in LD with SNP identified by PheGWAS
C – SNP identified by SNP thinning

Supplementary Table 1b: Summary of comparative analysis of loci identified by GLGC and PheGWAS results for association of loci with single traits

Loci identified by GLGC	88
Total Loci Detected by PheGWAS	88
Gene Match	77
Gene No Match	11
Gene not mapped	2
SNP's in category A	63
SNP's in category B	22
SNP's in category C	3

Supplementary Table 1c: LD Score between SNPs in GLGC and PheGWAS in single traits

Chromosome	Position MB	Trait	SNP Match	SNP ID	SNP ID phegwas	d'	r2
1	93.01	TC	M	rs7515577	rs1556562	1	1
1	182.17	HDL	M	rs1689800	rs1689797	0.99	0.89
2	135.84	TC	M	rs7570971	rs6730157	0.99	0.99
2	165.54	HDL	M	rs12328675	rs7607980	1	0.98
3	12.63	TC	NM	rs2290159	rs7616006	0.33	0.04
6	139.83	HDL	NM	rs605066	rs3861397	0.99	0.69
7	72.13	TG	NM	rs13238203	rs11974409	0.76	0.08
7	130.43	HDL	M	rs4731702	rs11765979	0.99	0.99
8	10.68	TG	NM	rs11776767	rs6995541	0.97	0.62
10	65.03	TG	M	rs10761731	rs10761762	1	0.8
11	18.63	TC	M	rs10128711	rs10832962	1	1
12	20.47	HDL	M	rs7134375	rs11045163	0.95	0.87
12	110.00	HDL	M	rs7134594	rs10850380	1	1
12	123.80	HDL	NM	rs4759375	rs2454722	0.2	0.2
12	125.26	HDL	M	rs838880	rs838876	0.94	0.84
15	44.25	TG	M	rs2929282	rs16948098	0.97	0.84
17	37.81	HDL	M	rs11869286	rs113612868	1	1
17	76.40	HDL	NM	rs4129767	rs4969178	0.94	0.57
18	57.85	HDL	M	rs12967135	rs6567160	1	1
19	8.43	HDL	M	rs7255436	rs2278236	1	0.99
19	49.21	TC	M	rs492602	rs516246	0.99	0.99
19	54.79	HDL	M	rs386000	rs103294	0.99	0.9
20	34.15	TC	NM	rs2277862	rs72664396	0.32	0.0002
22	21.93	HDL	NM	rs181362	rs113359481	NA	NA
22	38.55	TG	NM	rs5756931	rs3761445	0.73	0.49

Supplementary Table 2a: Comparative analysis of loci identified by GLGC and PheGWAS results for association of loci with multiple traits

Nearest Gene	SNP ID	Chromosome	Position MB	Multiple Traits	In PheGWAS	Gene M	Identifying GLGC SNP's
LDLRAP1	rs12027135	1	25.78	TC,LDL	DETECTED	NM	B
PCSK9	rs2479409	1	55.50	LDL,TC	DETECTED	M	B
ANGPTL3	rs2131925	1	63.03	TG,LDL,TC	DETECTED	M	B
SORT1	rs629301	1	109.82	LDL,TC	DETECTED	M	B
MOSC1	rs2642442	1	220.97	TC,LDL	DETECTED	NM	B
GALNT2	rs4846914	1	230.30	HDL,TG	DETECTED	M	B
IRF2BP2	rs514230	1	234.86	TC,LDL	DETECTED	NM	B
PIGV	rs12748152	1	27.14	HDL, LDL, TG	DETECTED	M	A
APOB	rs1367117	2	21.2600	LDL,TC	DETECTED	M	B
GCKR	rs1260326	2	27.7300	TG,TC	DETECTED	M	B
IRS1	rs2972146	2	227.1000	HDL,TG	DETECTED	NA	B
INSIG2	rs10490626	2	118.84	LDL, TCa	DETECTED	M	B
ABCG5/8	rs4299376	2	44.0700	LDL,TC	DETECTED	M	B
LOC84931	rs2030746	2	121.31	LDL, TC	DETECTED	NM	A
UGT1A1	rs11563251	2	234.68	TC, LDL	DETECTED	M	A
DNAJC13	rs17404153	3	132.16	LDL, HDLb	DETECTED	M	C
CMTM6	rs7640978	3	32.53	LDL, TC	DETECTED	M	A
LRPAP1	rs6831256	4	3.47	TG, TCa, LDLa	DETECTED	M	A
HMGCR	rs12916	5	74.6600	TC,LDL	DETECTED	M	A
TIMD4	rs6882076	5	156.3900	TC,TG,LDL	DETECTED	M	A
CSNK1G3	rs4530754	5	122.86	LDL, TC	DETECTED	M	A
LPA	rs1564348	6	160.5800	LDL,TC	DETECTED	M	B
RSPO3	rs1936800	6	127.44	HDL, TGa	DETECTED	M	B
HLA	rs3177928	6	32.4100	TC,LDL	DETECTED	M	B
FRK	rs9488822	6	116.3100	TC,LDL	DETECTED	M	B
MYLIP	rs3757354	6	16.1300	LDL,TC	DETECTED	M	A
HFE	rs1800562	6	26.0900	LDL,TC	DETECTED	M	A
VEGFA	rs998584	6	43.76	TG, HDL	DETECTED	M	A
MIR148A	rs4722551	7	25.99	LDL, TGc, TC	DETECTED	M	B
NPC1L1	rs2072183	7	44.5800	TC,LDL	DETECTED	M	B
MLXIPL	rs17145738	7	72.9800	TG,HDL	DETECTED	M	B
DNAH11	rs12670798	7	21.6100	TC,LDL	DETECTED	M	A
PPP1R3B	rs9987289	8	9.1800	HDL,TC,LDL	DETECTED	NM	B
LPL	rs12678919	8	19.8400	TG,HDL	DETECTED	M	B
TRIB1	rs2954029	8	126.4900	TG,TC,LDL,HDL	DETECTED	M	B
NAT2	rs1495741	8	18.2700	TG,TC	DETECTED	M	B
CYP7A1	rs2081687	8	59.3900	TC,LDL	DETECTED	M	B
PLEC1	rs11136341	8	145.0400	LDL,TC	DETECTED	M	B
SOX17	rs10102164	8	55.42	LDL, TC	DETECTED	M	A
TTC39B	rs581080	9	15.3100	HDL,TC	DETECTED	M	B
ABO	rs9411489	9	136.1550	LDL,TC	DETECTED	M	B
ABCA1	rs1883025	9	107.6600	HDL,TC	DETECTED	M	A
VLDLR	rs3780181	9	2.64	TC, LDL	DETECTED	M	A
GPAM	rs2255141	10	113.9300	TC,LDL	DETECTED	M	B
8-Mar	rs970548	10	46.01	HDL, TC	DETECTED	M	B
APOA1	rs964184	11	116.6500	TG,TC,HDL,LDL	DETECTED	M	B
ST3GAL4	rs11220462	11	126.2400	LDL,TC	DETECTED	M	B
FADS1-2-3	rs174546	11	61.5700	TG,LDL,TC,HDL	DETECTED	M	B
UBASH3B	rs7941030	11	122.5200	TC,HDL	DETECTED	M	B
LRP1	rs11613352	12	57.7900	TG,HDL	DETECTED	NM	B
BRAP	rs11065987	12	112.0700	TC,LDL	DETECTED	M	B
HNF1A	rs1169288	12	121.4200	TC,LDL	DETECTED	M	B
ZNF664	rs4765127	12	124.4600	HDL,TG	DETECTED	M	B
LIPC	rs1532085	15	58.6800	HDL,TC,TG	DETECTED	M	C (level 1)
FTO	rs1121980	16	53.81	HDL, TGb	DETECTED	M	C (level 1)
CETP	rs3764261	16	56.9900	HDL,LDL,TC,TG	DETECTED	M	B
HPR	rs2000999	16	72.1100	TC,LDL	DETECTED	M	A
OSBPL7	rs7206971	17	45.4300	LDL,TC	DETECTED	NM	B
DLG4	rs314253	17	7.09	TC, LDL	DETECTED	M	A
LIPG	rs7241918	18	47.1600	HDL,TC	DETECTED	M	B
APOE	rs4420638	19	45.4200	LDL,TC,HDL	DETECTED	M	B
LDLR	rs6511720	19	11.2000	LDL,TC	DETECTED	M	A
CILP2	rs10401969	19	19.4100	TC,TG,LDL	DETECTED	NM	A
PEPD	rs731839	19	33.9	TG, HDL	DETECTED	M	A
MAFB	rs2902940	20	39.0900	TC,LDL	DETECTED	NM	C(level 5)
TOP1	rs6029526	20	39.6700	LDL,TC	DETECTED	M	B
PLTP	rs6065906	20	44.5500	HDL,TG	DETECTED	M	B
HNF4A	rs1800961	20	43.0400	HDL,TC	DETECTED	M	A
PPARA	rs4253772	22	46.63	TC, LDLa	DETECTED	M	B

In this table, the columns in pheGWAS, gene match and SNP match corresponds to the PheGWAS results we obtained.

Match (M); if the gene symbol in a particular PheGWAS base pair interval segment is the same trait as reported in the GLGC
No Match (NM); if the gene is not identified by PheGWAS.

A - SNP identical with GLGC and PheGWAS
B – GLGC SNP in LD with SNP identified by PheGWAS
C – SNP identified by SNP thinning

Supplementary Table 2b: Summary of comparative analysis of loci identified by GLGC and PheGWAS results for association of loci with multiple traits

Loci identified by GLGC	69
Total Loci Detected by PheGWAS	69
Gene Match	59
Gene No Match	9
Gene not mapped	1
SNP’s in Category A	21
SNP’s in Category B	44
SNP’s in Category C	4

Supplementary Table 2c: LD Score between SNPs in GLGC and PheGWAS in multiple traits

Chromosome	Position MB	Multiple Traits	SNP Match	SNP ID	SNP ID phegwas	d'	r2
1	25.78	TC,LDL	M	rs12027135	rs11802413,rs10903129	1,1	0.98,0.98
1	55.50	LDL,TC	NM	rs2479409	rs11591147,rs11591147	1,1	0.01,0.01
1	63.03	TG,LDL,TC	M	rs2131925	rs4587594,rs11485618,rs3850634	1,1,1	0.99,0.98
1	109.82	LDL,TC	M	rs629301	rs646776,rs646776	1,1	0.99,0.99
1	220.97	TC,LDL	M	rs2642442	rs2642438,rs2642438	0.92,0.92	0.80,0.80
1	230.30	HDL,TG	M	rs4846914	rs1321257	0.99	0.95
1	234.86	TC,LDL	M	rs514230	rs558971,rs2587534	0.97,0.97	0.94,0.93
2	21.2600	LDL,TC	PM	rs1367117	rs515135	0.82	0.07
2	27.7300	TG,TC	M	rs1260326	rs780093,rs780093	0.95,0.95	0.91,0.91
2	227.1000	HDL,TG	M	rs2972146	rs1515110	0.94	0.88
2	118.84	LDL, TCa	M	rs10490626	rs17526895	1	0.99
2	44.0700	LDL,TC	M	rs4299376	rs6544713,rs6544713	0.99,0.99	0.97,0.97
3	132.16	LDL, HDLb	PM	rs17404153	NA	NA	NA
6	160.5800	LDL,TC	M	rs1564348	rs11753995	0.98	0.95
6	127.44	HDL, TGa	M	rs1936800	rs719726	0.95	0.75
6	32.4100	TC,LDL	PM	rs3177928	rs9391858,rs10947332	0.96,0.89	0.85,0.56
6	116.3100	TC,LDL	NM	rs9488822	rs11153594,rs6909746	0.95,0.95	0.66,0.66
7	25.99	LDL, TGc, TC	PM	rs4722551	rs4719841	0.88	0.1
7	44.5800	TC,LDL	NM	rs2072183	rs2073547,rs2073547	0.96,0.96	0.73,0.73
7	72.9800	TG,HDL	PM	rs17145738	rs11974409	0.99	0.55
8	9.1800	HDL,TC,LDL	M	rs9987289	rs4240624	1	1
8	19.8400	TG,HDL	PM	rs12678919	rs13702	0.99	0.31
8	126.4900	TG,TC,LDL,HDL	M	rs2954029	rs2954022,rs10808546	1,0.98	0.98,0.88
8	18.2700	TG,TC	PM	rs1495741	rs4921914,rs1961456	1,0.94	1,0.70
8	59.3900	TC,LDL	M	rs2081687	rs4738684,rs13277801	0.97,0.97	0.93,0.94
8	145.0400	LDL,TC	M	rs11136341	rs7832643,rs7832643	0.92,0.92	0.78,0.78
9	15.3100	HDL,TC	PM	rs581080	rs686030	0.96	0.62
9	136.1550	LDL,TC	M	rs9411489	rs579459,rs579459	1,1	0.83,0.83
10	113.9300	TC,LDL	M	rs2255141	rs2419604	0.99	0.97
11	116.6500	TG,TC,HDL,LDL	PM	rs964184	rs10790162	0.99	0.51
11	126.2400	LDL,TC	M	rs11220462	rs10893499	1	0.99
11	61.5700	TG,LDL,TC,HDL	M	rs174546	rs174535,rs174583,rs1535,rs102275	1,0.99,0.99,1	0.97,0.92,0.97,0.93
11	122.5200	TC,HDL	M	rs7941030	rs7117842,rs7117842	0.99,0.99	0.95,0.95
12	57.7900	TG,HDL	M	rs11613352	rs3741414	1	0.99
12	112.0700	TC,LDL	M	rs11065987	rs653178,rs653178	0.97,0.97	0.78,0.78
12	121.4200	TC,LDL	M	rs1169288	rs2244608	0.99	0.96
12	124.4600	HDL,TG	M	rs4765127	rs7973683,rs11057408	0.99,1	0.97,1
15	58.6800	HDL,TC,TG	NM	rs1532085	rs10468017,rs10468017,rs588136	0.98,0.98,0.001	0.67,0.67,0
16	53.81	HDL, TGb	PM	rs1121980	rs9930333	NA	NA
16	56.9900	HDL,LDL,TC,TG	PM	rs3764261	rs9989419,rs247616,rs247616,rs1800775	0.81,0.99,0.99,1	0.18,0.97,0.97,0.43
17	45.4300	LDL,TC	M	rs7206971	rs6504872,rs6504872	1,1	1,1
18	47.1600	HDL,TC	M	rs7241918	rs4939883,rs2156552	0.99,0.98	0.92,0.94
19	45.4200	LDL,TC,HDL	NM	rs4420638	rs7254892,rs7412,rs2075650	0.84,0.92,0.72	0.01,0.01,0.31
20	39.0900	TC,LDL	NM	rs2902940	rs2235367,rs6065311	0.02,0.02	0.0001,0.0001
20	39.6700	LDL,TC	PM	rs6029526	rs2235367,rs6065312	0.99,1	0.96,0.22
20	44.5500	HDL,TG	PM	rs6065906	rs4465830,rs4810479	0.99,1	0.99,0.63
22	46.63	TC, LDLa	M	rs4253772	rs4253776	0.99	0.95

Supplementary Table 3: PheGWAS findings showcasing Chromosome Number, Marker Name, Associated Traits, Genes and the respective p-values for Chromosome 1

Chr	Position group (Mb)	Marker Name	Associated Traits	Genes	P-Values	-log10 (P-value)
1	23	rs1077514	TOTAL_CHOLESTROL	ASAP3 RP4-654C18.1 TCEA3	6.4e-09	8.19
1	25	rs10903129 rs11802413	LDL TOTAL_CHOLESTROL	AL031284.1 RHCE RP3-469D22.1 TMEM57	3.03e-17 1.576e-14	16.52 13.8
1	26	rs2229714	HDL	MIR1976 RN7SL679P RPS6KA1 Y_RNA	6.094e-09	8.22
1	27	rs12748152 rs12748152 rs12748152	HDL LDL TRIGS	ARID1A PIGV RN7SL165P RN7SL501P ZDHHC18	9.737e-16 3.209e-12 1.1e-09	15.01 11.49 8.96
1	39	rs2296173 rs16837533	HDL TRIGS	BMP8A KIAA0754 MACF1 RP11-416A14.1 RP11-420K8.1	2.803e-17 3.873e-08	16.55 7.41
1	40	rs4660293 rs17513135	HDL TRIGS	BMP8A OXCT2P1 PABPC4 PPIEL RP11-69E11.4 RP11-69E11.8 SNORA55	2.863e-18 1.633e-08	17.54 7.79
1	55	rs11591147 rs11591147	LDL TOTAL_CHOLESTROL	BSND PCSK9 RP11-12C17.2 TMEM61 USP24	8.58e-143 8.827e-86	142.07 85.05
1	56	rs12066643	LDL	RP11-466L17.1 RP11-90C4.1	1.063e-08	7.97
1	62	rs1168013 rs1168032 rs1168013	LDL TRIGS TOTAL_CHOLESTROL	DOCK7 RP11-293K19.1	5.713e-33 1.486e-80 1.544e-80	32.24 79.83 79.81
1	63	rs11485618 rs4587594 rs3850634	LDL TRIGS TOTAL_CHOLESTROL	AL138847.1 ANGPTL3 DOCK7 RP5-849H19.2 RP11-230B22.1	3.729e-33 3.503e-82 1.39e-81	32.43 81.46 80.86
1	92	rs4970712 rs6603981	LDL TOTAL_CHOLESTROL	EVI5 GFI1	2.458e-13 7.846e-15	12.61 14.11
1	93	rs12133576 rs1556562 rs1556562	HDL LDL TOTAL_CHOLESTROL	DR1 RP4-713B5.2 RP4-717I23.3 Y_RNA EVI5 RP4-593M8.1	6.15e-11 4.558e-13 1.398e-14	10.21 12.34 13.85
1	109	rs12740374 rs646776 rs646776	HDL LDL TOTAL_CHOLESTROL	CELSR2 MYBPHL PSRC1 SARS SORT1	1.687e-15 1.63e-272 4.77e-187	14.77 271.79 186.32
1	110	rs333947 rs650985 rs518076	HDL LDL TOTAL_CHOLESTROL	CSF1 RP11-195M16.1 RP11-195M16.3 AC000032.2 AMPD2 GNAT2 GSTM1 GSTM2 GSTM4 RP5-1160K1.1 RP5-1160K1.6 GNAI3 GPR61 MIR197 RNU6V RP5-1160K1.3 RP5-1160K1.8	3.166e-09 1.899e-15 5.058e-10	8.5 14.72 9.3
1	150	rs267733	LDL	ANXA9 CERS2 FAM63A PRUNE RNU6-884P RP11-316M1.12 RP11-316M1.3 SETDB1	5.285e-09	8.28
1	156	rs12145743	HDL	CRABP2 HDGF ISG20L2 MRPL24 PRCC RP11-66D17.3 RP11-66D17.5 RRNAD1	1.803e-08	7.74
1	178	rs4650994	HDL	C1ORF220 C1orf220 LINC00083 RNA5SP69 TEX35	6.696e-09	8.17
1	182	rs1689797	HDL	GS1-122H1.2 GS1-122H1.3	2.852e-21	20.54
1	220	rs2642438 rs2642438 rs2642438	HDL LDL TOTAL_CHOLESTROL	HLA-AS1 MARC1 MARC2 RNU6ATAC35P RP11-295M18.2 RP11-295M18.6	7.781e-14 7.318e-16 1.283e-18	13.11 15.14 17.89
1	221	rs2247213 rs11590084	LDL TOTAL_CHOLESTROL	HLA-AS1 HLX RP11-295M18.2 RNU6ATAC35P RP11-295M18.6	1.412e-08 8.387e-08	7.85 7.08
1	230	rs4846914 rs1321257	HDL TRIGS	GALNT2	3.513e-41 5.986e-31	40.45 30.22
1	234	rs2587534 rs558971	LDL TOTAL_CHOLESTROL	RP4-781K5.4 RP4-781K5.5 RP4-781K5.6 RP4-781K5.7 RP4-781K5.8	8.055e-25 7.025e-28	24.09 27.15

Supplementary Table 4: PheGWAS findings showcasing Chromosome Number, Marker Name, Associated Traits, Genes and the respective p-values for Chromosome 2

Chr	Position group (Mb)	Marker Name	Associated Traits	Genes	P-Values	-log10 (P-value)
2	20	rs11679386 rs11679386	LDL TOTAL_CHOLESTROL	C2orf43	4.746e-14 2.226e-11	13.32 10.65
2	21	rs676210 rs1367117 rs676210 rs515135	HDL LDL TRIGS TOTAL_CHOLESTROL	APOB RP11-116D2.1	2.345e-54 9.48e-183 3.284e-71 6.38e-151	53.63 182.02 70.48 150.2
2	27	rs780093 rs1260326 rs780093	LDL TRIGS TOTAL_CHOLESTROL	AC109829.1 FNDC4 GCKR IFT172 RNU6-986P	2.359e-08 2.29e-239 2.591e-42	7.63 238.64 41.59
2	28	rs13030345 rs13030345	TRIGS TOTAL_CHOLESTROL	AC110084.1 MRPL33 RBKS	1.019e-57 2.787e-10	56.99 9.55
2	43	rs10495907 rs10495907	LDL TOTAL_CHOLESTROL	ABCG5 DYNC2LI1 PLEKHH2 RN7SKP66	1.264e-13 3.085e-11	12.9 10.51
2	44	rs6544713 rs6544713	LDL TOTAL_CHOLESTROL	ABCG5 ABCG8 DYNC2LI1 LRPPRC RNU6-1048P	4.843e-83 1.685e-81	82.31 80.77
2	62	rs1534420	LDL	AC092155.4 EHBP1	3.896e-08	7.41
2	63	rs2710642	LDL	EHBP1 RP11-443F16.1	6.089e-09	8.22
2	118	rs10490626 rs17526895	LDL TOTAL_CHOLESTROL	INSIG2 AC009303.1 CCDC93 RN7SL111P	1.697e-12 5.777e-09	11.77 8.24
2	121	rs2030746 rs2030746	LDL TOTAL_CHOLESTROL	AC073257.1 AC073257.2	8.605e-09 3.603e-08	8.07 7.44
2	135	rs16831243 rs6730157	LDL TOTAL_CHOLESTROL	CCNT2 MAP3K19 RAB3GAP1 SNORA40 ZRANB3	9.063e-12 1.179e-13	11.04 12.93
2	136	rs4988235 rs4988235	LDL TOTAL_CHOLESTROL	AC011893.3 AC011999.1 LCT MCM6 Y_RNA	3.218e-11 3.975e-14	10.49 13.4
2	165	rs7607980 rs10195252 rs13389219	HDL LDL TRIGS	AC019181.3 COBLL1 SNORA70F GRB14	1.807e-15 3.812e-08 2.598e-15	14.74 7.42 14.59
2	169	rs2287623 rs2287623	LDL TOTAL_CHOLESTROL	ABCB11	5.399e-08 4.085e-12	7.27 11.39
2	203	rs934287 rs11694172	LDL TOTAL_CHOLESTROL	ICA1L KRT8P15 WDR12 AC009960.3 AC009960.6 AC009960.7 FAM117B MTND4P30	1.214e-07 1.951e-09	6.92 8.71
2	204	rs1174604	TOTAL_CHOLESTROL	RAPH1	3.115e-07	6.51
2	211	rs1047891	HDL	CPS1	8.73e-10	9.06
2	216	rs1250229 rs1250229	LDL TOTAL_CHOLESTROL	AC012462.1 AC012462.2 AC012462.3 FN1	3.13e-08 2.382e-07	7.5 6.62
2	227	rs1515110 rs2972146	HDL TRIGS		8.038e-18 2.974e-15	17.09 14.53
2	234	rs11563251 rs11563251	LDL TOTAL_CHOLESTROL	AC114812.10 AC114812.5 AC114812.9 DNAJB3 MROH2A RPL17P11 UGT1A1 UGT1A10 UGT1A2P UGT1A3 UGT1A4 UGT1A5 UGT1A6 UGT1A7 UGT1A8 UGT1A9	4.499e-08 1.266e-09	7.35 8.9

Supplementary Table 5: PheGWAS findings showcasing Chromosome Number, Marker Name, Associated Traits, Genes and the respective p-values for Chromosome 3

Chr	Position group (Mb)	Marker Name	Associated Traits	Genes	P-Values	-log10 (P-value)
3	11	rs2606736	HDL	ATG7	4.799e-08	7.32
3	12	rs2292101 rs9875338 rs10440120 rs7616006	HDL LDL TRIGS TOTAL_CHOLESTROL	PPARG GSTM5P1 TSEN2 SYN2	1.359e-07 2.21e-11 5.343e-11 8.406e-17	6.87 10.66 10.27 16.08
3	32	rs7640978 rs7640978	LDL TOTAL_CHOLESTROL	AC104306.1 AC104306.2 CMTM6 CMTM7 DYNC1LI1	9.837e-09 1.658e-08	8.01 7.78
3	47	rs2290547	HDL	CCDC12 NBEAL2 NRADDP SETD2	3.69e-09	8.43
3	49	rs7613875	HDL	CTD-2330K9.2 CTD-2330K9.3 MON1A MST1R RBM6	1.792e-11	10.75
3	50	rs2013208	HDL	RBM5 RBM6 RP11-493K19.3	8.916e-12	11.05
3	52	rs13326165	HDL	NISCH NT5DC2 PBRM1 SMIM4 STAB1 TNNC1	9.042e-11	10.04
3	53	rs2336725	HDL	AC096887.1 RFT1 RP11-894J14.5 SERBP1P3 SFMBT1	2.316e-07	6.64
3	58	rs13315871	TOTAL_CHOLESTROL	PDHB PXK RP11-802O23.3	3.48e-08	7.46
3	119	rs6805251	HDL	GSK3B NR1I2 PHBP8	1.332e-08	7.88
3	131	rs13076253	HDL	CPNE4 MIR5704	4.961e-09	8.3
3	132	rs17404153 rs17404153	LDL TOTAL_CHOLESTROL	DNAJC13 NIP7P2	1.832e-09 1.362e-07	8.74 6.87
3	135	rs687339 rs645040	HDL TRIGS	MSL2 PCCB RP11-463H24.1	7.108e-13 1.83e-12	12.15 11.74
3	136	rs1279840 rs12630999	HDL TRIGS	PCCB STAG1 HMGN1P10 RNY4P4	2.135e-12 3.151e-10	11.67 9.5
3	142	rs4683438	TOTAL_CHOLESTROL	PAQR9 PCOLCE2 RP11-372E1.4 RP11-372E1.6 RP11-372E1.7 U2SURP	2.509e-07	6.6
3	156	rs1482852	HDL	LEKR1 LINC00880 LINC00881 RP11-6F2.5	6.34e-08	7.2
3	170	rs10513688	TRIGS	RNU1-70P SLC2A2	1.543e-07	6.81

Supplementary Table 6: PheGWAS findings showcasing Chromosome Number, Marker Name, Associated Traits, Genes and the respective p-values for Chromosome 4

Chr	Position group (Mb)	Marker Name	Associated Traits	Genes	P-Values	-log10 (P-value)
4	3	rs6818397 rs6831256 rs6818397	LDL TRIGS TOTAL_CHOLESTROL	DOK7 HGFAC RGS12 AL590235.1 LRPAP1 RP11-529E10.6	1.677e-08 1.602e-12 9.51e-11	7.78 11.8 10.02
4	26	rs10019888	HDL		4.901e-08	7.31
4	69	rs176813	TOTAL_CHOLESTROL	RP11-1267H10.4	1.178e-07	6.93
4	87	rs3775228 rs3736669	HDL TRIGS	AFF1	4.53e-09 6.542e-15	8.34 14.18
4	88	rs442177 rs442177	HDL TRIGS	AFF1	2.193e-09 1.316e-18	8.66 17.88
4	89	rs3822072 rs13133548	HDL TRIGS	FAM13A	4.058e-12 2.108e-07	11.39 6.68
4	100	rs2602836	HDL	ADH4 ADH5 METAP1 RP11-571L19.8 RP11-696N14.1	4.964e-08	7.3
4	102	rs17199964	HDL	BANK1	1.843e-07	6.73
4	103	rs13107325	HDL	SLC39A8	1.065e-15	14.97
4	157	rs6822892	HDL	PDGFC RP11-154F14.2	1.933e-07	6.71

Supplementary Table 7: PheGWAS findings showcasing Chromosome Number, Marker Name, Associated Traits, Genes and the respective p-values for Chromosome 5

Chr	Position group (Mb)	Marker Name	Associated Traits	Genes	P-Values	-log10 (P-value)
5	53	rs6450176	HDL	ARL15 RN7SL801P	6.875e-10	9.16
5	55	rs3936511 rs9686661	HDL TRIGS	AC022431.2 AC022431.3	2.959e-09 2.541e-16	8.53 15.59
5	67	rs4976033	HDL	CTC-537E7.3 CTD-2582M21.1	6.421e-08	7.19
5	74	rs12916 rs12916	LDL TOTAL_CHOLESTROL	COL4A3BP CTD-2235C13.1 CTD-2235C13.2 CTD-2235C13.3 HMGCR	7.792e-78 4.547e-74	77.11 73.34
5	75	rs2112347 rs2112347	LDL TOTAL_CHOLESTROL	ANKDD1B POC5 RNU6-680P SLC25A5P9	4.43e-30 1.72e-33	29.35 32.76
5	122	rs4530754 rs4530754	LDL TOTAL_CHOLESTROL	CSNK1G3 Y_RNA	3.576e-12 1.678e-09	11.45 8.78
5	156	rs6882076 rs6882076 rs6882076	LDL TRIGS TOTAL_CHOLESTROL	APOOP1 TIMD4	3.31e-31 1.513e-15 5.354e-41	30.48 14.82 40.27
5	173	rs72812840	TRIGS	C5orf47 CPEB4	2.896e-07	6.54

Supplementary Table 8: PheGWAS findings showcasing Chromosome Number, Marker Name, Associated Traits, Genes and the respective p-values for Chromosome 6

Chr	Position group (Mb)	Marker Name	Associated Traits	Genes	P-Values	-log10 (P-value)
6	16	rs3757354 rs3757354	LDL TOTAL_CHOLESTROL	MIR4639 MRPL42P2 MYLIP RP1-13D10.2 RP1-13D10.3 RP1-13D10.5 U3	2.087e-17 2.215e-15	16.68 14.65
6	25	rs17526722 rs1408272 rs1408272	HDL LDL TOTAL_CHOLESTROL	HIST1H2APS2 SLC17A2 SLC17A3 TRIM38 SLC17A1	1.42e-07 3.675e-09 5.034e-08	6.85 8.43 7.3
6	26	rs1800562 rs1800562	LDL TOTAL_CHOLESTROL	HFE HIST1H1C HIST1H1T HIST1H2AC HIST1H2BB HIST1H2BC HIST1H3C HIST1H4C U91328.2	8.253e-14 1.905e-12	13.08 11.72
6	29	rs5013093	TRIGS	HCG4P7 HCG4P8 HLA-G HLA-H HLA-T MICF MICG RPL7AP7	1.358e-07	6.87
6	30	rs1634721 rs1634721	TRIGS TOTAL_CHOLESTROL	HCG22 MUC21 MUC22	4.015e-13 5.279e-12	12.4 11.28
6	31	rs2844513 rs10947207 rs2247056 rs2247056	HDL LDL TRIGS TOTAL_CHOLESTROL	AL671883.1 FGFR3P1 HCP5 HLA-S MICA XXbac-BPG181B23.4 XXbac-BPG181B23.6 XXbac-BPG181B23.7 Y_RNA ZDHHC20P2 DHFRP2 HLA-B RNU6-283P XXbac-BPG248L24.12 HLA-C RPL3P2 USP8P1 WASF5P XXbac-BPG248L24.10 XXbac-BPG248L24.13	2.465e-09 2.448e-12 3.861e-21 2.203e-20	8.61 11.61 20.41 19.66
6	32	rs1980493 rs10947332 rs11752643 rs9391858	HDL LDL TRIGS TOTAL_CHOLESTROL	BTNL2 C6orf10 HCG23 HLA-DRA RNU6-603P HLA-DQA2 HLA-DQB1 HLA-DQB1-AS1 HLA-DQB2 HLA-DQB3 MIR3135B MTCO3P1 XXbac-BPG254F23.6 XXbac-BPG254F23.7 HNRNPA1P2	3.764e-10 6.969e-18 3.96e-19 7.204e-22	9.42 17.16 18.4 21.14
6	34	rs205262 rs3800457 rs2814982	HDL LDL TOTAL_CHOLESTROL	C6orf106 RP3-391O22.2 RP3-391O22.3 SPDEF RN7SL200P RP11-140K17.2 RP11-140K17.3 RP3-375P9.2 RP3-391O22.1 snoU13 PACSIN1	3.878e-13 2.3e-07 3.678e-15	12.41 6.64 14.43
6	35	rs3822921 rs3800406 rs3800406	HDL LDL TOTAL_CHOLESTROL	AL138721.1 ANKS1A TCP11 SCUBE3	2.349e-09 9.392e-08 1.014e-13	8.63 7.03 12.99
6	39	rs2758886 rs2758886	LDL TOTAL_CHOLESTROL	KCNK16 KCNK17 KIF6	1.845e-07 3.008e-08	6.73 7.52
6	43	rs998584 rs998584	HDL TRIGS	RP1-261G23.7 VEGFA	2.269e-11 3.424e-15	10.64 14.47
6	109	rs884366	HDL	C6orf183 CCDC162P PTCHD3P3 RP11-425D10.1 snoU13	1.671e-07	6.78
6	116	rs6909746 rs11153594	LDL TOTAL_CHOLESTROL	FRK TPI1P3	7.86e-11 1.266e-14	10.1 13.9
6	127	rs1936800 rs719726	HDL TRIGS	RSPO3	3.055e-10 2.486e-08	9.51 7.6
6	135	rs9376090	TOTAL_CHOLESTROL	CTA-212D2.2 HBS1L	2.595e-09	8.59
6	139	rs3861397 rs634869	HDL TRIGS	RP11-12A2.3	8.401e-11 1.776e-14	10.08 13.75
6	152	rs12525163	HDL	ESR1	1.524e-07	6.82
6	160	rs9457931 rs1564348 rs2665357 rs11753995	HDL LDL TRIGS TOTAL_CHOLESTROL	LPA LPAL2 IGF2R RP11-288H12.4 RP11-317M22.1 SLC22A1 SLC22A2 AL591069.1 SLC22A3	7.297e-13 2.762e-21 8.327e-10 1.839e-23	12.14 20.56 9.08 22.74
6	161	rs1084651 rs10455872 rs10455872	HDL LDL TOTAL_CHOLESTROL	LPA PLG RP1-81D8.3 RP1-81D8.4	1.347e-11 1.936e-15 7.236e-17	10.87 14.71 16.14

Supplementary Table 9: PheGWAS findings showcasing Chromosome Number, Marker Name, Associated Traits, Genes and the respective p-values for Chromosome 7

Chr	Position group (Mb)	Marker Name	Associated Traits	Genes	P-Values	-log10 (P-value)
7	1	rs1997243 rs1997243	HDL TOTAL_CHOLESTROL	AC073957.15 AC091729.7 AC091729.8 C7orf50 GPER1 GPR146 MIR339 RP11-449P15.1	2.27e-07 2.719e-10	6.64 9.57
7	6	rs702485	HDL	DAGLB KDELR2 RAC1	6.45e-12	11.19
7	16	rs17286602	HDL	AC006035.1 ISPD RP11-196O16.1	2.932e-07	6.53
7	17	rs4142995	HDL	SNX13	9.365e-12	11.03
7	21	rs12670798 rs12670798	LDL TOTAL_CHOLESTROL	DNAH11	4.812e-14 9.478e-17	13.32 16.02
7	25	rs4722551 rs4719841 rs4722551	LDL TRIGS TOTAL_CHOLESTROL	CTD-2227E11.1 MIR148A	3.949e-14 8.864e-11 7.019e-09	13.4 10.05 8.15
7	44	rs2073547 rs2073547	LDL TOTAL_CHOLESTROL	AC004938.5 DDX56 NPC1L1 RNU6-1097P TMED4	1.923e-21 3.83e-21	20.72 20.42
7	50	rs4917014	HDL	AC020743.4 IKZF1	1.026e-08	7.99
7	71	rs2909969	TRIGS	TYW1B	1.528e-07	6.82
7	72	rs17145738 rs11974409	HDL TRIGS	BAZ1B BCL7B MLXIPL TBL2	4.952e-13 1.36e-100	12.31 99.87
7	73	rs17145750 rs17145750	HDL TRIGS	MLXIPL TBL2	5.654e-12 1.03e-99	11.25 98.99
7	116	rs38855	TRIGS	MET	2.109e-08	7.68
7	130	rs11765979 rs287621	HDL TRIGS	KLF14 RP11-36B6.1	3.111e-17 7.671e-09	16.51 8.12
7	150	rs17173637	HDL	AOC1 RP5-1051J4.4 TMEM176A TMEM176B	1.899e-08	7.72

Supplementary Table 10: PheGWAS findings showcasing Chromosome Number, Marker Name, Associated Traits, Genes and the respective p-values for Chromosome 8

Chr	Position group (Mb)	Marker Name	Associated Traits	Genes	P-Values	-log10 (P-value)
8	8	rs2976940	TRIGS	CTA-398F10.1 CTA-398F10.2 SGK223	1.138e-08	7.94
8	9	rs4240624 rs9987289 rs9693857 rs9987289	HDL LDL TRIGS TOTAL_CHOLESTROL	RNU6-1151P RNU6-526P RP11-115J16.1 RP11-115J16.2 RP11-115J16.3 RP11-375N15.1	1.323e-45 8.529e-24 1.691e-08 1.844e-36	44.88 23.07 7.77 35.73
8	10	rs7014168 rs6995541	HDL TRIGS	CTD-2135J3.3 PINX1 SNORD112 SOX7 RP11-177H2.2	9.203e-10 1.343e-12	9.04 11.87
8	11	rs1062219	TRIGS	C8orf49 FDFT1 GATA4 NEIL2 RP11-297N6.4 SUB1P1	1.686e-09	8.77
8	18	rs1961456 rs4921914 rs1961456	LDL TRIGS TOTAL_CHOLESTROL	NAT2 NATP RP11-685B14.1 RP11-685B14.3	6.883e-08 4.867e-17 3.458e-16	7.16 16.31 15.46
8	19	rs13702 rs12678919	HDL TRIGS	LPL	1.28e-160 1.82e-199	159.89 198.74
8	55	rs10102164 rs10102164	LDL TOTAL_CHOLESTROL	RP11-53M11.2 RP11-53M11.3 RP11-53M11.4 SOX17	3.742e-11 4.596e-11	10.43 10.34
8	59	rs13277801 rs4738684 rs4738684	LDL TRIGS TOTAL_CHOLESTROL	CYP7A1 RP11-114M5.1 UBXN2B RP11-114M5.3	3.994e-17 8.819e-09 1.116e-23	16.4 8.05 22.95
8	116	rs2293889 rs2737252 rs2737252	HDL LDL TOTAL_CHOLESTROL	TRPS1	4.271e-17 7.039e-14 1.634e-16	16.37 13.15 15.79
8	121	rs4871137	HDL	RP11-713M15.2 SNTB1	1.926e-07	6.72
8	126	rs10808546 rs2954029 rs2954022 rs2954029	HDL LDL TRIGS TOTAL_CHOLESTROL	RN7SL590P RP11-136O12.2 TRIB1	4.106e-30 2.095e-50 2.23e-113 2.421e-65	29.39 49.68 112.65 64.62
8	144	rs10087900 rs6984820 rs6984820	HDL LDL TOTAL_CHOLESTROL	GLI4 GPIHBP1 ZFP41 EPPK1 MIR661 PLEC	2.174e-09 3.769e-09 1.634e-07	8.66 8.42 6.79
8	145	rs7832643 rs7832643	LDL TOTAL_CHOLESTROL	GRINA MIR661 PARP10 PLEC	2.671e-17 3.121e-13	16.57 12.51

Supplementary Table 11: PheGWAS findings showcasing Chromosome Number, Marker Name, Associated Traits, Genes and the respective p-values for Chromosome 9

Chr	Position group (Mb)	Marker Name	Associated Traits	Genes	P-Values	-log10 (P-value)
9	2	rs3780181 rs3780181	LDL TOTAL_CHOLESTROL	RP11-125B21.2 VLDLR	1.764e-09 6.668e-10	8.75 9.18
9	15	rs686030 rs686030 rs581080	HDL TRIGS TOTAL_CHOLESTROL	RN7SL157P TTC39B	4.292e-27 2.226e-07 1.022e-13	26.37 6.65 12.99
9	16	rs3927680	LDL	BNC2	2.15e-07	6.67
9	19	rs10757056	TOTAL_CHOLESTROL	ACER2 DENND4C NDUFA5P3 RP11-363E7.4 RP11-513M16.7 RP11-513M16.8 RPS6	5.361e-08	7.27
9	107	rs1883025 rs1883025 rs1883025 rs1883025	HDL LDL TRIGS TOTAL_CHOLESTROL	ABCA1 RP11-217B7.2	1.496e-65 6.141e-11 2.908e-07 5.749e-53	64.83 10.21 6.54 52.24
9	136	rs579459 rs579459	LDL TOTAL_CHOLESTROL	ABO LCN1P2 RP11-430N14.4 SURF6 Y_RNA	2.419e-44 8.828e-42	43.62 41.05

Supplementary Table 12: PheGWAS findings showcasing Chromosome Number, Marker Name, Associated Traits, Genes and the respective p-values for Chromosome 10

Chr	Position group (Mb)	Marker Name	Associated Traits	Genes	P-Values	-log10 (P-value)
10	5	rs1832007	TRIGS	AKR1C4 AKR1CL1 RP11-445P17.3 RP11-445P17.6 U8	1.718e-12	11.76
10	17	rs10904908 rs10904908	LDL TOTAL_CHOLESTROL	RP11-124N14.3 TRDMT1 VIM VIM-AS1	3.135e-07 2.601e-11	6.5 10.58
10	45	rs10900221 rs10900221	HDL TOTAL_CHOLESTROL	ALOX5 MARCH8 RP11-67C2.2	1.004e-09 7.963e-09	9 8.1
10	46	rs970548 rs970548	HDL TOTAL_CHOLESTROL	MARCH8	1.706e-10 8.431e-09	9.77 8.07
10	64	rs7073746 rs7073746	HDL TRIGS	JMJD1C NRBF2 RNU6-543P RP11-144G16.1	1.791e-08 1.234e-15	7.75 14.91
10	65	rs10761771 rs10761762	HDL TRIGS	JMJD1C JMJD1C-AS1 PRELID1P3 RP11-351O1.3	4.12e-09 1.061e-17	8.39 16.97
10	94	rs8211 rs2068888	HDL TRIGS	CYP26A1 CYP26C1 EXOC6 NIP7P1 RP11-348J12.2	8.049e-08 1.682e-11	7.09 10.77
10	113	rs2250802 rs2419604 rs2250802 rs2255141	HDL LDL TRIGS TOTAL_CHOLESTROL	GPAM	2.022e-17 7.49e-14 1.21e-10 6.514e-16	16.69 13.13 9.92 15.19
10	114	rs2148489 rs2148489	HDL TOTAL_CHOLESTROL	GUCY2GP TECTB	1.412e-10 2.366e-10	9.85 9.63
10	115	rs7076938	HDL	ADRB1	1.712e-07	6.77

Supplementary Table 13: PheGWAS findings showcasing Chromosome Number, Marker Name, Associated Traits, Genes and the respective p-values for Chromosome 11

Chr	Position group (Mb)	Marker Name	Associated Traits	Genes	P-Values	-log10 (P-value)
11	10	rs2923084	HDL	AMPD3 RNU6ATAC33P	5.02e-08	7.3
11	14	rs2303975	HDL	RP11-21L19.1 RRAS2 SPON1	1.585e-07	6.8
11	18	rs10832962 rs10832962	LDL TOTAL_CHOLESTROL	RP11-137N23.1 RP11-504G3.4 SPTY2D1 SPTY2D1-AS1 UEVLD	6.619e-14 1.539e-14	13.18 13.81
11	45	rs74458891	HDL	CTD-2210P24.1 CTD-2210P24.2 CTD-2210P24.3	6.492e-09	8.19
11	46	rs3136441 rs6485690	HDL TRIGS	ARHGAP1 ATG13 CKAP5 F2 MIR5582 SNORD67 ZNF408	6.759e-29 2.061e-07	28.17 6.69
11	47	rs3847502 rs10501321 rs1681630	HDL TRIGS TOTAL_CHOLESTROL	MADD MYBPC3 NR1H3 RP11-17G12.3 SPI1 ACP2 DDB2 PTPRJ RP11-793I11.1	3.306e-38 1.412e-08 6.929e-08	37.48 7.85 7.16
11	48	rs7946766 rs4752805	HDL TOTAL_CHOLESTROL	AC103828.1 PTPRJ	4.967e-24 1.617e-09	23.3 8.79
11	49	rs11040329	HDL	CTD-2026G22.1	5.46e-12	11.26
11	50	rs2201637	HDL	RP11-227P3.1 RP11-347H15.1	5.273e-10	9.28
11	51	rs11246602	HDL	OR4C46 OR4C50P OR4C7P OR4R2P	1.681e-10	9.77
11	55	rs11229606	HDL	OR4A10P OR4A13P OR4A14P OR4A17P OR4A50P OR4A9P OR4X7P RP11-131J4.12	3.117e-10	9.51
11	56	rs6591323	HDL	OR5AL1 OR5AL2P OR5M4P OR5M9 OR5R1 OR8L1P OR8U1	1.508e-08	7.82
11	61	rs102275 rs174583 rs174535 rs1535	HDL LDL TRIGS TOTAL_CHOLESTROL	DAGLA FADS1 FADS2 FEN1 MIR1908 MIR611 MYRF RP11-467L20.10 TMEM258 FADS3	6.404e-28 7.003e-41 1.733e-41 8.624e-39	27.19 40.15 40.76 38.06
11	65	rs12801636	HDL	AP001362.1 EHBP1L1 FAM89B KCNK7 MAP3K11 MIR4489 MIR4690 PCNXL3 RELA SIPA1 SSSCA1	3.147e-08	7.5
11	75	rs499974	HDL	CTD-2530H12.1 CTD-2530H12.2 DGAT2 MOGAT2 RN7SL786P	1.12e-08	7.95
11	116	rs964184 rs964184 rs10790162 rs964184	HDL LDL TRIGS TOTAL_CHOLESTROL	AP006216.10 AP006216.11 AP006216.5 APOA4 APOA5 BUD13 ZNF259	6.086e-48 2.008e-26 1.1e-249 2.838e-55	47.22 25.7 248.96 54.55
11	117	rs508487 rs508487 rs508487 rs508487	HDL LDL TRIGS TOTAL_CHOLESTROL	PAFAH1B2 PCSK7 RNF214 SIDT2 TAGLN	5.45e-11 1.773e-10 9.22e-119 1.002e-27	10.26 9.75 118.04 27
11	118	rs11603023	TOTAL_CHOLESTROL	AP002954.3 ARCN1 IFT46 PHLDB1 RNU6-1157P TREH	1.151e-08	7.94
11	122	rs7117842 rs7117842	HDL TOTAL_CHOLESTROL	GLULP3 RP11-266E8.2 UBASH3B	1.057e-14 2.479e-15	13.98 14.61
11	126	rs17135399 rs10893499 rs11220462	HDL LDL TOTAL_CHOLESTROL	DCPS RP11-712L6.5 ST3GAL4 TIRAP KIRREL3	4.26e-09 3.861e-21 5.487e-15	8.37 20.41 14.26

Supplementary Table 14: PheGWAS findings showcasing Chromosome Number, Marker Name, Associated Traits, Genes and the respective p-values for Chromosome 12

Chr	Position group (Mb)	Marker Name	Associated Traits	Genes	P-Values	-log10 (P-value)
12	9	rs4883201	TOTAL_CHOLESTROL	A2ML1 KLRG1 M6PR PHC1	1.742e-09	8.76
12	20	rs11045163	HDL		3.196e-09	8.5
12	21	rs4149056	TRIGS	RP11-125O5.2 SLCO1B1	1.782e-07	6.75
12	50	rs10876041	TOTAL_CHOLESTROL	DIP2B LARP4	1.028e-07	6.99
12	57	rs3741414 rs11613352	HDL TRIGS	AC126614.1 ARHGAP9 GLI1 INHBC INHBE MARS R3HDM2 RNU6-879P RP11-756H6.1	6.095e-14 9.397e-14	13.22 13.03
12	107	rs10861661	TRIGS	RFX4 RIC8B RP11-144F15.1 RP11-482D24.3	2.598e-07	6.59
12	109	rs2241210 rs12321904	HDL TOTAL_CHOLESTROL	KCTD10 MMAB UBE3B MVK RNU4-32P	2.489e-20 2.077e-08	19.6 7.68
12	110	rs10850380 rs10850380	HDL TOTAL_CHOLESTROL	MMAB MVK RNU4-32P UBE3B	4.058e-19 2.561e-08	18.39 7.59
12	111	rs3184504 rs3184504 rs3184504	HDL LDL TOTAL_CHOLESTROL	AC002395.1 ATXN2 RP3-473L9.4 SH2B3	4.097e-12 4.203e-12 1.623e-17	11.39 11.38 16.79
12	112	rs653178 rs11065987 rs653178	HDL LDL TOTAL_CHOLESTROL	ATXN2 RP11-686G8.1 RP11-686G8.2 U7 BRAP PCNPP1	1.064e-12 1.204e-11 1.038e-16	11.97 10.92 15.98
12	113	rs12580178	LDL	RPH3A	2.307e-08	7.64
12	121	rs1169288 rs2244608	LDL TOTAL_CHOLESTROL	AC079602.1 C12orf43 HNF1A HNF1A-AS1 OASL RP11-216P16.2	6.447e-21 9.618e-18	20.19 17.02
12	122	rs11057405 rs895953	HDL TRIGS	CLIP1 RP11-512M8.3 RP11-512M8.5 VPS33A AC084018.1 HPD RHOF RP11-347I19.8 RP11-7M8.2 SETD1B TMEM120B	1.231e-09 8.578e-08	8.91 7.07
12	123	rs2454722 rs10773003	HDL TOTAL_CHOLESTROL	HCAR1 HCAR2 HCAR3 RP11-324E6.6 RP11-324E6.9 C12orf65 CDK2AP1 MPHOSPH9 RNA5SP375 RP11-282O18.3 RP11-282O18.6 RP11-282O18.7 SBNO1	3.308e-14 4.083e-09	13.48 8.39
12	124	rs7973683 rs11057408	HDL TRIGS	CCDC92 DNAH10 DNAH10OS FAM101A RP11-214K3.18 RP11-214K3.19 RP11-214K3.20 RP11-214K3.21 RP11-214K3.22 RP11-214K3.23 RP11-214K3.5 RP11-380L11.3 RP11-380L11.4 ZNF664	5.259e-14 2.049e-12	13.28 11.69
12	125	rs838876	HDL	SCARB1	7.325e-33	32.14
12	133	rs12423664	TRIGS	FBRSL1 MUC8 RP11-503G7.1 RP11-503G7.2	8.189e-08	7.09

Supplementary Table 15: PheGWAS findings showcasing Chromosome Number, Marker Name, Associated Traits, Genes and the respective p-values for Chromosome 13

Chr	Position group (Mb)	Marker Name	Associated Traits	Genes	P-Values	-log10 (P-value)
13	32	rs4942486 rs4942486	LDL TOTAL_CHOLESTROL	BRCA2 IFIT1P1 N4BP2L1 RP11-298P3.4 SNORA16	2.261e-11 7.084e-08	10.65 7.15

Supplementary Table 16: PheGWAS findings showcasing Chromosome Number, Marker Name, Associated Traits, Genes and the respective p-values for Chromosome 14

Chr	Position group (Mb)	Marker Name	Associated Traits	Genes	P-Values	-log10 (P-value)
14	24	rs8017377 rs6573778	LDL TOTAL_CHOLESTROL	CBLN3 KHNYN NFATC4 NYNRIN SDR39U1	2.516e-15 2.958e-11	14.6 10.53
14	105	rs4983559	HDL	AKT1 CTD-3051D23.1 CTD-3051D23.4 LINC00638 RP11-982M15.2 RPS26P49 RPS2P4 SIVA1 ZBTB42	9.565e-09	8.02

Supplementary Table 17: PheGWAS findings showcasing Chromosome Number, Marker Name, Associated Traits, Genes and the respective p-values for Chromosome 15

Chr	Position group (Mb)	Marker Name	Associated Traits	Genes	P-Values	-log10 (P-value)
15	41	rs721772	HDL	ITPKA LTK RPAP1 TCEB1P2 TYRO3	2.358e-07	6.63
15	42	rs2412710 rs2412710	HDL TRIGS	CAPN3 GANC RNU6-188P RP11-164J13.1 ZNF106	1.36e-09 1.658e-11	8.87 10.78
15	43	rs486301	TRIGS	CKMT1B MAP1A PPIP5K1 TP53BP1	5.816e-08	7.24
15	44	rs492571 rs16948098	HDL TRIGS	FRMD5 PIN4P1	1.265e-12 4.838e-17	11.9 16.32
15	58	rs10468017 rs588136 rs10468017	HDL TRIGS TOTAL_CHOLESTROL	ALDH1A2 LIPC RP11-355N15.1	1.21e-188 3.365e-30 7.232e-48	187.92 29.47 47.14
15	59	rs424346	HDL	ADAM10 RN7SKP95 RP11-123C21.1 RP11-123C21.2 RP11-30K9.6 RP11-30K9.7 U3	4.84e-08	7.32
15	63	rs2652834 rs2652834	HDL TRIGS	LACTB RP11-244F12.2 RP11-69G7.1 RPS27L TPM1	3.592e-11 1.918e-08	10.44 7.72
15	72	rs1035744	TRIGS	CELF6 PARP6 PKM RP11-106M3.2 RP11-106M3.3	1.448e-07	6.84
15	73	rs2415168	TRIGS	ADPGK ADPGK-AS1	2.775e-07	6.56

Supplementary Table 18: PheGWAS findings showcasing Chromosome Number, Marker Name, Associated Traits, Genes and the respective p-values for Chromosome 16

Chr	Position group (Mb)	Marker Name	Associated Traits	Genes	P-Values	-log10 (P-value)
16	15	rs3198697	TRIGS	MIR1972-1 NTAN1 PDXDC1 RP11-680G24.4 RP11-680G24.5 RRN3	2.207e-08	7.66
16	30	rs11649653	TRIGS	AC106782.20 AC135048.13 BCL7C CTF1 CTF2P FBXL19 FBXL19-AS1 MIR4519 MIR762 ORAI3	1.56e-07	6.81
16	31	rs749671	TRIGS	AC135050.1 AC135050.5 BCKDK KAT8 PRSS53 RP11-196G11.1 RP11-196G11.2 RP11-196G11.4 STX4 VKORC1 ZNF646 ZNF668	6.106e-10	9.21
16	53	rs1121980 rs9930333	HDL TRIGS	FTO	6.791e-09 3.251e-08	8.17 7.49
16	56	rs9989419 rs247616 rs1800775 rs247616	HDL LDL TRIGS TOTALCHOLESTROL	AC012181.1 CETP HERPUD1 NLRC5 RP11-325K4.2 RP11-325K4.3 RPS24P17 SLC12A3	0 2.566e-37 1.332e-26 4.47e-32	329.32 36.59 25.88 31.35
16	57	rs59207181 rs1532624 rs7205804 rs1532624	HDL LDL TRIGS TOTALCHOLESTROL	AC012181.1 CETP HERPUD1 NLRC5 RP11-322D14.1 RP11-325K4.2 RP11-325K4.3	0 1.844e-26 3.431e-25 1.39e-29	329.32 25.73 24.46 28.86
16	67	rs16942887 rs3809630	HDL TOTALCHOLESTROL	AC040162.1 CENPT CTC-479C5.10 CTC-479C5.11 CTC-479C5.12 CTC-479C5.17 CTRL EDC4 LCAT NRN1L NUTF2 PSKH1 PSMB10 SLC12A4 THAP11 RANBP10 TSNAXIP1	8.281e-54 1.544e-08	53.08 7.81
16	68	rs16957696 rs13816	HDL TOTALCHOLESTROL	AC130462.1 CTC-479C5.6 DDX28 DPEP2 DPEP3 DUS2 KARSP3 RNU6-359P SLC12A4	1.31e-46 8.945e-08	45.88 7.05
16	71	rs8053891 rs8053891	LDL TOTALCHOLESTROL	ATP5A1P3 DHODH IST1 PKD1L3 RP11-498D10.5 RP11-498D10.6 RP11-498D10.8 RPL39P31	3.76e-18 2.628e-18	17.43 17.58
16	72	rs2000999 rs2000999	LDL TOTALCHOLESTROL	DHODH DHX38 HP HPR PMFBP1 RP11-384M15.3 TXNL4B	4.219e-41 6.804e-41	40.38 40.17
16	81	rs2925979 rs2925979	HDL TRIGS	CMIP RP11-391L3.5	1.321e-19 2.136e-07	18.88 6.67

Supplementary Table 19: PheGWAS findings showcasing Chromosome Number, Marker Name, Associated Traits, Genes and the respective p-values for Chromosome 17

Chr	Position group (Mb)	Marker Name	Associated Traits	Genes	P-Values	-log10 (P-value)
17	4	rs8069974	TOTAL_CHOLESTROL	ARRB2 CXCL16 GLTPD2 MED11 PLD2 PSMB6 RP11-314A20.5 RP11-81A22.5 TM4SF5 VMO1 ZMYND15	7.795e-08	7.11
17	7	rs314253 rs314253	LDL TOTAL_CHOLESTROL	ACADVL ASGR1 DLG4 DVL2 MIR324 PHF23 RPL7AP64	3.436e-10 2.808e-10	9.46 9.55
17	8	rs4791641	LDL	AURKB CTC1 LINC00324 PFAS RANGRF RP11-849F2.10 RP11-849F2.8 RP11-849F2.9 SLC25A35	1.314e-07	6.88
17	29	rs2854322	TOTAL_CHOLESTROL	AK4P1 NF1 RAB11FIP4	2.898e-07	6.54
17	37	rs113612868 rs9972882	HDL TOTAL_CHOLESTROL	AC087491.2 ERBB2 PGAP3 PNMT PPP1R1B STARD3 TCAP NEUROD2	5.447e-20 4.158e-08	19.26 7.38
17	38	rs8069176 rs12309	HDL TOTAL_CHOLESTROL	GSDMB IKZF3 LRRC3C ORMDL3 RP11-387H17.4 ZPBP2 CSF3 GSDMA MED24 PSMD3 RP11-387H17.6 SNORD124 THRA	5.93e-13 1.953e-07	12.23 6.71
17	41	rs231492 rs8077889	HDL TRIGS	CD300LG FAM215A MPP2 PPY RP11-527L4.2 RP11-527L4.5 RP11-527L4.6 C17orf105 DUSP3 MPP3 RP5-905N1.2 SOST	2.002e-07 9.879e-09	6.7 8.01
17	45	rs6504872 rs6504872	LDL TOTAL_CHOLESTROL	CTD-2026D20.2 EFCAB13 ITGB3 RP11-290H9.4	3.479e-13 6.987e-12	12.46 11.16
17	64	rs1801689	LDL	APOH CEP112 PSMD7P1	9.809e-12	11.01
17	65	rs12602912	TRIGS	AC006534.2 BPTF	1.291e-07	6.89
17	66	rs4148005	HDL	ABCA8	5.743e-14	13.24
17	67	rs2886232 rs2886232	LDL TOTAL_CHOLESTROL	ABCA10 ABCA6	3.876e-11 3.874e-08	10.41 7.41
17	76	rs4969178	HDL	AC061992.1 AC061992.2 DNAH17 PGS1 RN7SL236P RP11-806H10.4 SNORA30 SOCS3	1.532e-12	11.81

Supplementary Table 20: PheGWAS findings showcasing Chromosome Number, Marker Name, Associated Traits, Genes and the respective p-values for Chromosome 18

Chr	Position group (Mb)	Marker Name	Associated Traits	Genes	P-Values	-log10 (P-value)
18	46	rs2156498	HDL	C18orf32 DYM MIR1539 RP11-110H1.1 RP11-110H1.4 RP11-110H1.9 RPL17 RPL17-C18orf32 SNORD58A SNORD58B SNORD58C SRP72P1	2.175e-07	6.66
18	47	rs4939883 rs2156552	HDL TOTAL_CHOLESTROL	LIPG RP11-813F20.1	1.796e-66 1.247e-31	65.75 30.9
18	57	rs6567160	HDL	RNU4-17P RP11-795H16.2 RP11-795H16.3 RPS3AP49	2.918e-09	8.53

Supplementary Table 21: PheGWAS findings showcasing Chromosome Number, Marker Name, Associated Traits, Genes and the respective p-values for Chromosome 19

Chr	Position group (Mb)	Marker Name	Associated Traits	Genes	P-Values	-log10 (P-value)
19	7	rs4804833 rs7248104	HDL TRIGS	AC010336.1 CTD-3193O13.1 CTD-3193O13.10 CTD-3193O13.11 CTD-3193O13.13 CTD-3193O13.8 CTD-3193O13.9 CTXN1 EVI5L LRRC8E MAP2K7 RN7SL115P RNA5SP463 SNAPC2 TGFBR3L TIMM44 INSR	9.891e-08 5.045e-10	7 9.3
19	8	rs2278236 rs4804311	HDL TRIGS	AC010323.1 ANGPTL4 CTD-2550O8.7 KANK3 MARCH2 MIR4999 NDUFA7 RAB11B RAB11B-AS1 RPS28 AC092316.1 AC092316.2 AC130469.2 ADAMTS10 MYO1F PRAM1 ZNF414	3.185e-18 1.485e-09	17.5 8.83
19	10	rs11881156 rs11881156	LDL TOTALCHOLESTROL	AC007229.3 C19orf38 CARM1 DNM2 MIR199A1 TMED1	1.701e-55 8.708e-44	54.77 43.06
19	11	rs737337 rs6511720 rs6511720	HDL LDL TOTALCHOLESTROL	C19orf80 CTC-510F12.2 DOCK6 KANK2 RN7SL298P LDLR SMARCA4 SPC24	4.564e-17 3.85e-262 5.43e-202	16.34 261.42 201.26
19	18	rs4808802	TOTALCHOLESTROL	AC010335.1 CTD-3137H5.1 ELL ISYNA1 SSBP4	3.266e-08	7.49
19	19	rs10401969 rs10401969 rs10401969	LDL TRIGS TOTALCHOLESTROL	AC138430.4 HAPLN4 MAU2 NCAN SUGP1 TM6SF2	2.654e-54 9.702e-70 4.126e-77	53.58 69.01 76.38
19	33	rs731839 rs731839	HDL TRIGS	AKR1B1P7 CEBPG PEPD	3.441e-09 2.651e-09	8.46 8.58
19	35	rs1688030	TRIGS	AC020907.1 CTD-2527I21.14 CTD-2527I21.9 FXYD3 GRAMD1A HPN HPN-AS1 SCN1B	1.99e-07	6.7
19	44	rs926054 rs926054	LDL TOTALCHOLESTROL	AC069278.4 CTC-512J12.4 CTC-512J12.6 ZNF180 ZNF229 ZNF285 ZNF285B	3.358e-15 2.407e-08	14.47 7.62
19	45	rs2075650 rs7254892 rs439401 rs7412	HDL LDL TRIGS TOTALCHOLESTROL	APOC1 APOC1P1 APOC4 APOC4-APOC2 APOE CTB-129P6.4 PVRL2 TOMM40 APOC2 CLPTM1 CTB-129P6.11	9.716e-26 0 1.423e-66 1.56e-283	25.01 329.32 65.85 282.81
19	46	rs7255743 rs7255743	LDL TOTALCHOLESTROL	ERCC1 FOSB OPA3 PPM1N RTN2 VASP	1.408e-25 9.61e-13	24.85 12.02
19	49	rs492602 rs516246	LDL TOTALCHOLESTROL	FUT1 FUT2 IZUMO1 MAMSTR NTN5 RASIP1 RN7SL345P SEC1P	9.422e-14 9.133e-17	13.03 16.04
19	50	rs1132990	TRIGS	CTD-3148I10.13 CTD-3148I10.15 CTD-3148I10.9 FCGRT FLT3LG MIR150 NOSIP RCN3 RPL13A RPS11 SNORD32A SNORD33 SNORD34 SNORD35A SNORD35B Y_RNA hsa-mir-150	6.125e-08	7.21
19	52	rs17695224	HDL	AC006272.1 AC006272.2 FPR1 FPR3 ZNF577	2.417e-13	12.62
19	54	rs103294 rs103294	HDL TOTALCHOLESTROL	AC008984.2 AC008984.5 AC008984.6 AC008984.7 AC010492.4 AC010492.5 AC010518.3 AC098789.1 CTD-2337J16.1 LILRA3 LILRA4 LILRA5 LILRB2 LILRB5 MIR4752 RNU6-1307P RPS9 VN1R104P	3.995e-30 2.27e-11	29.4 10.64

Supplementary Table 22: PheGWAS findings showcasing Chromosome Number, Marker Name, Associated Traits, Genes and the respective p-values for Chromosome 20

Chr	Position group (Mb)	Marker Name	Associated Traits	Genes	P-Values	-log10 (P-value)
20	12	rs364585	LDL	RP11-157E14.1 RP5-1069C8.2 RP5-1069C8.3 SPTLC3	4.278e-10	9.37
20	17	rs2328223	LDL	AL035045.1 RNU6-192P RP5-905G11.3	5.632e-09	8.25
20	33	rs6058202	HDL	AL121753.1 EDEM2 MMP24 MMP24-AS1 MT1P3 PROCR RNA5SP483 RP4-614O4.12	1.275e-07	6.89
20	34	rs7264396 rs7264396	LDL TOTAL_CHOLESTROL	C20orf173 ERGIC3 FER1L4 RP3-477O4.16 RP3-477O4.5 RPL36P4 RPL37P1 SPAG4	4.412e-08 6.585e-13	7.36 12.18
20	39	rs6065311 rs6029143 rs2235367	LDL TRIGS TOTAL_CHOLESTROL	PLCG1 RP1-1J6.2 TOP1 RP3-511B24.5 RP3-511B24.6 RPL23AP81 ZHX3	1.656e-30 4.933e-08 7.219e-25	29.78 7.31 24.14
20	40	rs4142393 rs4142393	LDL TOTAL_CHOLESTROL	CHD6 RP4-620E11.4	1.347e-09 2.743e-07	8.87 6.56
20	43	rs1800961 rs1800961 rs1800961	HDL LDL TOTAL_CHOLESTROL	AL132772.1 C20orf62 HNF4A MIR3646 RP5-1013A22.2 RP5-1013A22.5 RP5-881L22.4 RP5-881L22.6	1.639e-34 6.034e-10 1.342e-24	33.79 9.22 23.87
20	44	rs4465830 rs4810479	HDL TRIGS	FTLP1 PCIF1 PLTP ZNF335 CTSA NEURL2 RP3-337O18.9 SPATA25 ZSWIM1 ZSWIM3	5.175e-40 2.067e-34	39.29 33.68
20	45	rs6066141	TRIGS	EYA2 GAPDHP54	2.339e-08	7.63

Supplementary Table 23: PheGWAS findings showcasing Chromosome Number, Marker Name, Associated Traits, Genes and the respective p-values for Chromosome 22

Chr	Position group (Mb)	Marker Name	Associated Traits	Genes	P-Values	-log10 (P-value)
22	21	rs113359481 rs111562164	HDL TOTAL_CHOLESTROL	RIMBP3C RN7SKP221 SCARNA17 SCARNA18 UBE2L3 CCDC116 YDJC	4.303e-18 4.363e-10	17.37 9.36
22	30	rs5763662 rs5763662	LDL TOTAL_CHOLESTROL	AC003681.1 CTA-85E5.10 MTMR3	1.191e-08 7.719e-08	7.92 7.11
22	35	rs138777	TOTAL_CHOLESTROL	HMGXB4 MIR3909 RP3-510H16.3 TOM1	4.74e-08	7.32
22	38	rs3761445	TRIGS	AL021977.1 MAFF PLA2G6 RN7SL704P RP1-5O6.5 TMEM184B	8.062e-12	11.09
22	46	rs4253776 rs4253772	LDL TOTAL_CHOLESTROL	CDPF1 PKDREJ PPARA TTC38	3.353e-08 9.852e-09	7.47 8.01

FIGURES:

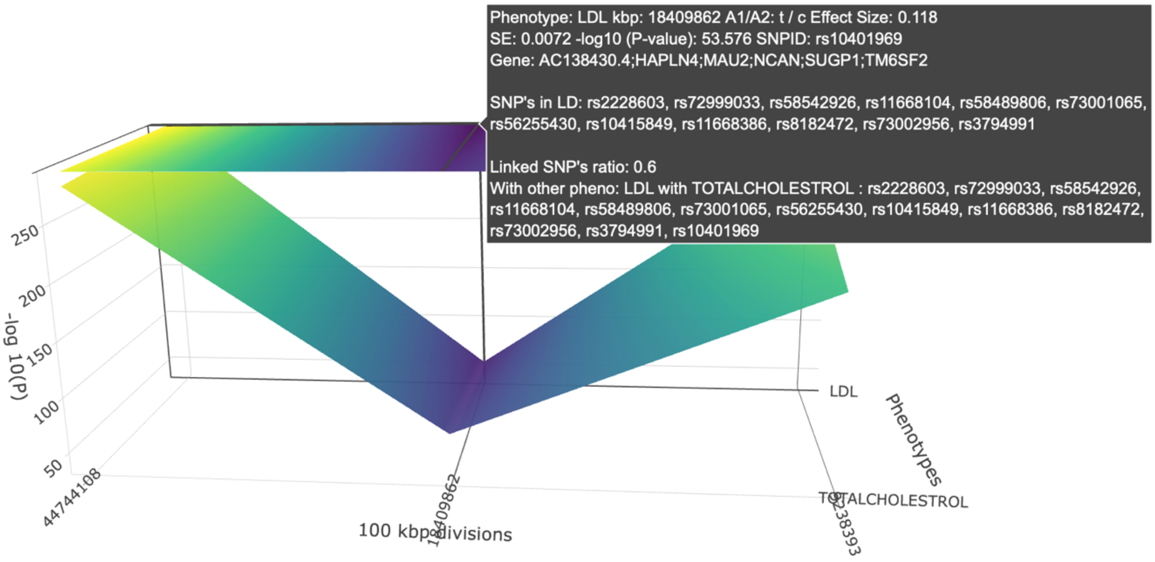

Fig 1: PheGWAS diagram for chromosome 19 on Total Cholesterol and LDL.

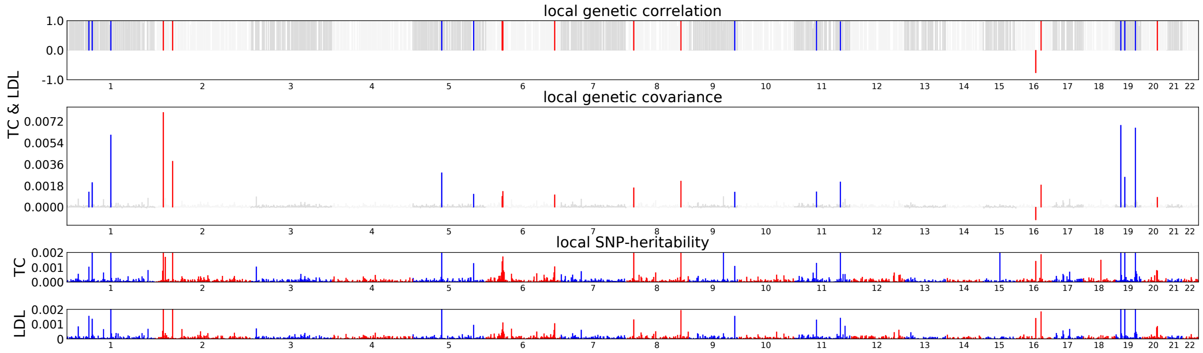

Fig 2: Estimates of Local Genetic Covariance for the Pairs of Traits LDL & TC using ρ-HESS. In 19th chromosome it shows 3 peaks for local genetic correlation.

Section 1: Colocalization analysis performed for chromosome 19 for BP region from 44744108 to 46102697.

Section 1a: Using HyprColoc

For each iteration of the algorithm, HyPrColoc returns:

i. a cluster of putatively colocalized traits
ii. the posterior probability that these traits are colocalized
iii. the 'regional association' probability* (which is always > the posterior probability)
iv. a candidate causal variant explaining the shared association
v. the proportion of the posterior probability explained by this variant (which represents the HyPrColoc multi-trait fine-mapping probability).

Following is the output:

```
Call:
hyprcoloc

Results:
  iteration                         traits posterior_prob regional_prob candidate_snp posterior_explained_by_snp dropped_trait
1         1 LDL_beta, TOTALCHOLESTROL_beta              1             1        rs7412                          1          <NA>
2         2                           None             NA             1          <NA>                         NA      HDL_beta
```

Section 1b: Using MOLOC

The output is a list with three elements:

1. First element is a data frame containing the priors, likelihoods and Posteriors for each locus and each combination. We usually care about the last columns, the posterior probability of a shared variant for each model;
2. Second element is the number of SNPs analyzed, in common across all datasets;
3. Third element of the output is a data frame with the posterior probability of the most likely SNP.

Following is the output.

```
> print(moloc[[1]])
         prior sumbf logBF_locus     PPA
a        1e-04    47          43 0.0e+00
a,b      1e-08  1746        1737 0.0e+00
a,c      1e-08   799         789 0.0e+00
a,d      1e-08   136         126 0.0e+00
a,bc     1e-09  2499        2489 3.3e-38
a,bd     1e-09  1815        1805 0.0e+00
a,cd     1e-09   867         857 0.0e+00
a,bcd    1e-10  2568        2558 1.5e-09
a,b,c    1e-12  2498        2488 8.4e-42
a,b,d    1e-12  1835        1826 0.0e+00
a,b,cd   1e-13  2566        2556 3.9e-13
a,c,d    1e-12   888         878 0.0e+00
a,bd,c   1e-13  2565        2555 7.8e-14
a,bc,d   1e-13  2588        2579 1.7e-03
a,b,c,d  1e-16  2588        2578 1.2e-06
b        1e-04  1700        1695 0.0e+00
b,c      1e-08  2452        2442 6.2e-58
b,d      1e-08  1788        1778 0.0e+00
ac,b     1e-09  2498        2488 1.1e-38
ad,b     1e-09  1816        1806 0.0e+00
b,cd     1e-09  2520        2510 2.7e-29
acd,b    1e-10  2566        2557 5.1e-10
b,c,d    1e-12  2541        2531 2.9e-23
ad,b,c   1e-13  2568        2558 3.0e-12
ac,b,d   1e-13  2588        2578 7.6e-04
c        1e-04   753         748 0.0e+00
c,d      1e-08   841         831 0.0e+00
ab,c     1e-09  2499        2489 1.7e-38
ad,c     1e-09   869         859 0.0e+00
bd,c     1e-09  2520        2510 3.8e-29
abd,c    1e-10  2567        2557 1.1e-09
ab,c,d   1e-13  2587        2578 6.6e-04
d        1e-04    88          83 0.0e+00
ab,d     1e-09  1835        1825 0.0e+00
ac,d     1e-09   888         878 0.0e+00
bc,d     1e-09  2541        2531 4.2e-20
abc,d    1e-10  2588        2578 1.0e+00
ab       1e-05  1747        1742 0.0e+00
ab,cd    1e-10  2567        2557 1.1e-09
ac       1e-05   800         795 0.0e+00
ac,bd    1e-10  2567        2557 1.1e-09
ad       1e-05   117         112 0.0e+00
ad,bc    1e-10  2569        2559 5.6e-09
bc       1e-05  2453        2448 2.0e-54
bd       1e-05  1768        1763 0.0e+00
cd       1e-05   821         816 0.0e+00
abc      1e-06  2500        2495 4.8e-35
abd      1e-06  1815        1810 0.0e+00
acd      1e-06   868         863 0.0e+00
bcd      1e-06  2521        2516 1.1e-25
abcd     1e-07  2568        2563 3.0e-06
zero     1e+00     0           0 0.0e+00
```

```
> print(moloc[[2]])
[1] 137
> print(moloc[[3]])
     coloc_ppas best.snp.coloc
a       1.0e+00         rs7412
b       1.0e+00         rs7412
ab      1.0e+00         rs7412
c       1.0e+00         rs7412
ac      7.7e-04         rs7412
bc      1.0e+00         rs7412
abc     1.0e+00         rs7412
d       3.1e-06         rs7412
ad      3.0e-06         rs7412
bd      3.0e-06         rs7412
abd     3.0e-06         rs7412
cd      3.0e-06         rs7412
acd     3.0e-06         rs7412
bcd     3.0e-06         rs7412
abcd    3.0e-06         rs7412
```
